# Supplementary material for: Myotubularin related protein-2 and its phospholipid substrate PIP2 control Piezo2-mediated mechanotransduction in peripheral sensory neurons
Source: eLife. 2018 Mar 9;7:e32346. doi: 10.7554/eLife.32346 (PMC5898911; doi:10.7554/eLife.32346)
Supplement: Supplementary file 1. — The table shows the displacement threshold and inactivation time constant (τ) values for all electrophysiological data presented in this study (please see Materials and methods for details on the calculation of each value). Values are represented as mean ± SEM and cell numbers are indicated by ‘n’. Data were not significant (ns) unless otherwise mentioned. Please note: for SA-MA currents, it was not possible to fit the current traces of all cells with a mono or bi-exponential fit (please see Materials and methods for details), hence the cell numbers measured for the inactivation time constant (τ) are lower than actual cell numbers measured and reported in Figure 2. [file elife-32346-supp1.docx]

| **Supplementary File 1** |  | Displacement threshold  (µm) | Inactivation time constant, τ (ms) |
| --- | --- | --- | --- |
| HEK293 cells | **Piezo2 + mock** | 3.47 ± 0.30  (n=17) | 3.29 ± 0.54  (n=11) |
|  | **Piezo2 + Mtmr2** | 4.92 ± 0.38  (n=12); p=0.0098; Mann-Whitney test | 3.57 ± 0.60  (n=11) |
|  |  |  |  |
|  | **Piezo2 + mock** | 4.59 ± 0.26  (n=17) | 2.56 ± 0.36  (n=14) |
|  | **Piezo2 + Mtmr2 C417S** | 3.84 ± 0.38  (n=18) | 2.89 ± 0.68  (n=14) |
|  |  |  |  |
|  | **Piezo1 + mock** | 3.64 ± 0.20  (n=25) | 12.00 ± 1.19  (n=19) |
|  | **Piezo1 + Mtmr2** | 3.85 ± 0.32  (n=20) | 17.16 ± 2.81  (n=12); p = 0.3011; Mann-Whitney test |
|  |  |  |  |
| HEK293 cells | **Piezo2-P1 mutant + mock** | 3.54 ± 0.39  (n=13) | 3.17 ± 0.40  (n=13) |
|  | **Piezo2-P1 mutant + Mtmr2** | 4.00 ± 0.4  (n=10) | 3.46 ± 0.88  (n=10) |
|  |  |  |  |
| DRG neurons | **CTRL**  **(Allstar negative control)** | 3.17 ± 0.18  (n=66) | 4.21 ± 0.28  (n=57) |
|  | **Mtmr2 siRNA** | 2.87 ± 0.17  (n=77) | 4.63 ± 0.27  (n=63) |
|  |  |  |  |
|  | **mock plasmid** | 3.71 ± 0.28  (n=28) | 3.67 ± 0.49  (n=23) |
|  | **Mtmr2 cDNA** | 3.90 ± 0.24  (n=30) | 3.59 ± 0.40  (n=27) |
|  |  |  |  |
| DRG neurons  DRG neurons  DRG neurons | **Mtmr2 siRNA + DMSO** | 2.15 ± 0.21  (n=27) | 4.75 ± 0.52  (n=22) |
|  | **Mtmr2 siRNA + Apilimod** | 3.10 ± 0.23  (n=30); p=0.005 (vs. Mtmr2 siRNA + DMSO); Kruskal Wallis trst followed by Dunn’s multiple comparison | 3.57 ± 0.39  (n=25); ns; Kruskal Wallis trst followed by Dunn’s multiple comparison |
|  | **Mtmr2 siRNA + Wortmannin** | 2.68 ± 0.21  (n=28) | 4.94 ± 0.70  (n=27) |
|  |  |  |  |
|  | **+ DMSO** | 2.81 ± 0.41  (n=16) | 5.44 ± 0.76  (n=14) |
|  | **+ Apilimod** | 2.08 ± 0.52  (n=12) | 6.47 ± 1.09  (n=8) |
|  |  |  |  |
|  | **Isotonic extra** | 3.59 ± 0.20  (n=53) | 3.13 ± 0.34  (n=35) |
|  | **Hypotonic extra** | 2.84 ± 0.14  (n=81); p=0.0021; Mann-Whitney test | 3.18 ± 0.24  (n=61) |
| DRG neurons | **Mtmr2 siRNA + Isotonic intra** | 3.59 ± 0.25  (n=29) | 2.96 ± 0.43  (n=23) |
|  | **Mtmr2 siRNA + Hypotonic intra** | 4.52 ± 0.21  (n=25); p=0.0131;  Mann-Whitney test | 3.05 ± 0.47  (n=21) |
|  |  |  |  |
|  | **Mtmr2 cDNA + Isotonic extra** | 3.50 ± 0.33  (n=14) | 4.43 ± 0.86  (n=9) |
|  | **Mtmr2 cDNA + Hypotonic extra** | 2.79 ± 0.27  (n=19) | 3.89 ± 0.56  (n=14) |
|  |  |  |  |
|  | **CTRL** | 3.74 ± 0.29  (n=27) | 3.49 ± 0.42  (n=20) |
|  | **+ 1µM PI(3)P** | 3.65 ± 0.32  (n=17) | 2.65 ± 0.36  (n=14); ns; Kruskal Wallis trst followed by Dunn’s multiple comparison |
|  | **+ 1µM PI(3,5)P_2_** | 3.52 ± 0.31  (n=21) | 2.32 ± 0.50  (n=9); ns; Kruskal Wallis trst followed by Dunn’s multiple comparison |
|  |  |  |  |
| DRG neurons | **CTRL (IA-MA)** | 3.17 ± 0.33  (n=23) | 16.28 ± 1.10  (n=23) |
|  | **Mtmr2 siRNA (IA-MA)** | 3.40 ± 0.53  (n=10) | 16.87 ± 2.94  (n=9) |
|  |  |  |  |
|  | **CTRL (SA-MA)** | 2.50 ± 0.32  (n=12) | 55.43 ± 5.18  (n=5) |
|  | **Mtmr2 siRNA (SA-MA)** | 2.36 ± 0.52  (n=11) | 48.60 ± 4.93  (n=3) |
