# Supplementary material for: Myotubularin related protein-2 and its phospholipid substrate PIP2 control Piezo2-mediated mechanotransduction in peripheral sensory neurons
Source: eLife. 2018 Mar 9;7:e32346. doi: 10.7554/eLife.32346 (PMC5898911; doi:10.7554/eLife.32346)
Supplement: Transparent reporting form [file elife-32346-transrepform.docx]

***eLife’s* transparent reporting form**

We encourage authors to provide detailed information *within their submission* to facilitate the interpretation and replication of experiments. Authors can upload supporting documentation to indicate the use of appropriate reporting guidelines for health-related research (see [EQUATOR Network](http://www.equator-network.org/%20)), life science research (see the [BioSharing Information Resource](https://biosharing.org/)), or the [ARRIVE guidelines](http://www.plosbiology.org/article/info:doi/10.1371/journal.pbio.1000412) for reporting work involving animal research. Where applicable, authors should refer to any relevant reporting standards documents in this form.

If you have any questions, please consult our Journal Policies and/or contact us: [editorial@elifesciences.org](mailto:editorial@elifesciences.org).

**Sample-size estimation**

- You should state whether an appropriate sample size was computed when the study was being designed
- You should state the statistical method of sample size computation and any required assumptions
- If no explicit power analysis was used, you should describe how you decided what sample (replicate) size (number) to use

Please outline where this information can be found within the submission (e.g., sections or figure legends), or explain why this information doesn’t apply to your submission:

No statistical method was used to determine sample sizes. Yet, our sample sizes conform to or exceed standards in the field.
For each data set the number of biological replicates (number of animals, number of analyzed cells), the number of independent cell culture preparations and the number of technical replicates (repetitions of lipid-peptide assays) are indicated in the respective figure legends, the respective graphs in each figure and in the respective method section.

**Replicates**

- You should report how often each experiment was performed
- You should include a definition of biological versus technical replication
- The data obtained should be provided and sufficient information should be provided to indicate the number of independent biological and/or technical replicates
- If you encountered any outliers, you should describe how these were handled
- Criteria for exclusion/inclusion of data should be clearly stated
- High-throughput sequence data should be uploaded before submission, with a private link for reviewers provided (these are available from both GEO and ArrayExpress)

Please outline where this information can be found within the submission (e.g., sections or figure legends), or explain why this information doesn’t apply to your submission:

For each data set the number of biological replicates (number of animals, number of analyzed cells, number of fibers; indicated by “n”), the number of independent cell culture preparations and the number of technical replicates (independent repetitions of lipid-peptide assays) are indicated in the respective figure legends, the respective graphs in each figure or in the respective method section.

Biological replicates are defined as:

- number of cells analyzed by electrophysiology, AFM or immunocytochemistry in HEK cells

- number of animals from which independent DRG cultures were prepared (for q-PCR)

- number of animals used for immunohistochemistry

Technical replicates are defined as:

- independent repetitions of lipid-peptide assays

- triplicate values for each qPCR experiment in each independently prepared DRG culture

Outlier analysis:

Details of outlier analysis are provided in the methods section (Statistics: lines 688-696). Outlier analysis was only performed on electrophysiological datasets. This was sometimes necessary due to the large variability seen in the recordings throughout the course of this study. Reasons for variability of recordings in DRG cultures are given in more detail in the methods sections (Electrophysiology: lines 554-561). Outlier analysis was carried out using the Grubb’s test followed by testing whether the outlier value exceeded ‘mean+3* standard deviation’. Outlier analysis was only performed on current values at maximal stimulation. Only if a value met both criteria (Grubb’s outlier and >’mean+3*standard deviation’) the cell was excluded from further analysis. Datasets, where a single outlier was removed: Figure 1h, Figure 2b, Figure 4b, Figure 4d, Figure 4-figure supplement 1a,c.

For all other datasets, all measured data were analyzed and are represented in the respective figures and figure legends.

**Statistical reporting**

- Statistical analysis methods should be described and justified
- Raw data should be presented in figures whenever informative to do so (typically when N per group is less than 10)
- For each experiment, you should identify the statistical tests used, exact values of N, definitions of center, methods of multiple test correction, and dispersion and precision measures (e.g., mean, median, SD, SEM, confidence intervals; and, for the major substantive results, a measure of effect size (e.g., Pearson's r, Cohen's d)
- Report exact p-values wherever possible alongside the summary statistics and 95% confidence intervals. These should be reported for all key questions and not only when the p-value is less than 0.05.

Please outline where this information can be found within the submission (e.g., sections or figure legends), or explain why this information doesn’t apply to your submission:

The details of statistical tests used for each experiment are provided in the Methods section of the manuscript (lines 676-700). Additionally, the statistical tests used for each experimental dataset are also indicated in the respective figure legends. The numerical data, number of biological and technical replicates and the results of the statistical analysis are also provided for each dataset.

In brief, for comparison of independent data between two groups, where tests of normality were not met, the Mann-Whitney test was used. For data sets where the data were normally distributed the unpaired t-test was used.

When more than two groups were compared, the Kruskal-Wallis test with Dunn’s multiple comparison test (non-normally distributed data) was used.

For qPCR data, the mRNA levels of sample were compared to a theoretical value of 1 (mRNA level of control) using the One sample t-test.

For analysis of stimulus-response curves in electrophysiology data 2-way ANOVA was used (compares responses between conditions at different stimulus magnitudes). Multiple comparisons test was performed using the Holm-Sidak’s method.

(For large datasets, or papers with a very large number of statistical tests, you may upload a single table file with tests, Ns, etc., with reference to sections in the manuscript.)

**Group allocation**

- Indicate how samples were allocated into experimental groups (in the case of clinical studies, please specify allocation to treatment method); if randomization was used, please also state if restricted randomization was applied
- Indicate if masking was used during group allocation, data collection and/or data analysis

Please outline where this information can be found within the submission (e.g., sections or figure legends), or explain why this information doesn’t apply to your submission:

Not applicable to this study.

**Additional data files (“source data”)**

- We encourage you to upload relevant additional data files, such as numerical data that are represented as a graph in a figure, or as a summary table
- Where provided, these should be in the most useful format, and they can be uploaded as “Source data” files linked to a main figure or table
- Include model definition files including the full list of parameters used
- Include code used for data analysis (e.g., R, MatLab)
- Avoid stating that data files are “available upon request”

Please indicate the figures or tables for which source data files have been provided:

We provide a Supplementary File 1, which reports additional electrophysiological parameters, i.e. displacement threshold and inactivation time constant (τ) values for all electrophysiological data presented in this study.
